# Identification and characterization of insect-specific proteins by genome data analysis

**DOI:** 10.1186/1471-2164-8-93

**Published:** 2007-04-04

**Authors:** Guojie Zhang, Hongsheng Wang, Junjie Shi, Xiaoling Wang, Hongkun Zheng, Gane Ka-Shu Wong, Terry Clark, Wen Wang, Jun Wang, Le Kang

**Affiliations:** 1 State Key Laboratory of Integrated Management of Pest Insects and Rodents, Institute of Zoology Chinese Academy of Sciences, Haidian Beijing 100080, China; 2 Beijing Institute of Genomics of Chinese Academy of Sciences, Beijing Genomics Institute, Beijing 101300, China; 3 CAS-Max Plank Junior Research Group, Key Laboratory of Cellular and Molecular Evolution, Kunming Institute of Zoology, Chinese Academy of Science (CAS), Kunming, Yunnan 650223, China; 4 Department of Electrical Engineering and Computer Science, The University of Kansas, 2001 Eaton Hall, Lawrence, KS 66044, USA; 5 Department of Biochemistry and Molecular Biology, University of Southern Denmark, DK-5230, Odense M, Denmark

## Abstract

**Background:**

Insects constitute the vast majority of known species with their importance including biodiversity, agricultural, and human health concerns. It is likely that the successful adaptation of the Insecta clade depends on specific components in its proteome that give rise to specialized features. However, proteome determination is an intensive undertaking. Here we present results from a computational method that uses genome analysis to characterize insect and eukaryote proteomes as an approximation complementary to experimental approaches.

**Results:**

Homologs in common to *Drosophila melanogaster*, *Anopheles gambiae*, *Bombyx mori, Tribolium castaneum*, and *Apis mellifera *were compared to the complete genomes of three non-insect eukaryotes (opisthokonts) *Homo sapiens*, *Caenorhabditis elegans *and *Saccharomyces cerevisiae*. This operation yielded 154 groups of orthologous proteins in *Drosophila *to be insect-specific homologs; 466 groups were determined to be common to eukaryotes (represented by three opisthokonts). ESTs from the hemimetabolous insect *Locust migratoria *were also considered in order to approximate their corresponding genes in the insect-specific homologs. Stress and stimulus response proteins were found to constitute a higher fraction in the insect-specific homologs than in the homologs common to eukaryotes.

**Conclusion:**

The significant representation of stress response and stimulus response proteins in proteins determined to be insect-specific, along with specific cuticle and pheromone/odorant binding proteins, suggest that communication and adaptation to environments may distinguish insect evolution relative to other eukaryotes. The tendency for low *Ka/Ks *ratios in the insect-specific protein set suggests purifying selection pressure. The generally larger number of paralogs in the insect-specific proteins may indicate adaptation to environment changes. Instances in our insect-specific protein set have been arrived at through experiments reported in the literature, supporting the accuracy of our approach.

## Background

Insects constitute nearly 80% of species on earth and are among the most diverse group of organisms in the history of life, giving them considerable potential to provide insight into evolutionary mechanisms. Insects, with their large number of species, their biomass, diversity of adaptation, and ecological impact, support the structure and function of ecosystem and biodiveristy on the lands of the earth. Numerous crops rely on insects for pollination, with the importance of insects extending into other agricultural and human health concerns. Insects have been in existence for at least 400 million years, making them among the earliest land animals. Though nearly one million insect species have been classified and named, their actual number is believed to be between 2.5 and 10 million. It is widely accepted that insects diverged as members of one of the largest subphyla in arthropods more than 390 million years ago. During this time, insects experienced rapid evolution and a radiation that is considered faster than any other group [1], migrating into nearly all available environmental niches except the benthic zone [2]. Mitochondrial DNA strongly supports an insect-crustacean clade as a sister group, which excludes the other arthropod subphyla collectively known as the myriapods [3]. The insects are a monophyletic group, a universally held view supported by morphological and molecular features.

The structure of an organism is an outgrowth of development tailored to meet functional demands in an idiosyncratic evolutionary history. Like other segmented animals, insects are composed of a series of repeated units called metameres. Extant arthropods share many taxonomical characteristics, such as an exoskeleton, jointed appendages, and reduced coeloms and hemocoels. The segments of the insect body are organized into three major tagmata unique to this subclass: the head, thorax, and abdomen [4]. The thorax has three pair of legs, and in pterygotes, the wings. In the abdomen, we find the presence of an ovipositor in females. In addition to the macro-scale features mentioned above, other defining features of the Insecta include: the loss of musculature and the presence of the Johnsonton's organ in the antenna, loss of articulations between the coxae and the sterna, sub-segmentation of the tarsus into units called tarsomeres, articulation of the pretarsal claws with the apical-most tarsomere [5], and the presence, at least primitively, of a long terminal filament [6]. Insects are one of only four lineages of animals with powered flight, the others being pterosaurs, birds, and bats. Wings refine insect design, vastly improving mobility, dispersal, and complex behaviors to adapt to environmental challenges. It is widely held that insects evolved flight just once, at least 100 million years before pterosaurs, perhaps 170 million years ago [5]. Other noteworthy features include the development of the posterior tentorium into a tranverse bar, and metamorphism and segmentation of metameres [7,8].

It is likely that the specialized features of the Insecta clade are based on components specific to its proteome. Characterization of this protein set should improve understanding of the molecular basis for the diversification of insects and their extensive success in ecological niches. Toward elucidating this molecular basis, we have characterized the eukaryote and insect proteomes. The large number of eukaryote genome sequences now available, including various insect genomes, makes it possible to characterize proteomes computationally. In this work, we utilized the insect genome sequences of fruit fly, mosquito, silk worm, beetle, honeybee, locust ESTs, and the non-insect eukaryote genomes of nematode, human, and yeast. (The insect-species in our study cover *holometabolous *and *hemimetabolous *development.) Since our approach utilizes genome sequence for approximating the proteome, the resolution of the proteome characterization improves as more genomes become available. This rapid characterization of proteomes through computation facilitates rational hypothesis generation and experiment design in applied research in many areas, such as biodiversity, agriculture and human health.

## Results

### Insect and Eukaryote protein sets

We modeled the insect proteome by selecting the subset of *Drosophila *protein sequences with homology to predicted genes in all insect-species studied here. Similarly, we defined the subset in *Drosophila *common to the eukaryote species studied here: mosquito, silkworm, beetle, honeybee, human, nematode and yeast. Because at this time it is not possible to definitively determine the eukaryote and insect proteomes, estimates are useful for comparative assessments. Our protein sets were derived from a collection of 13,525 protein sequences established for *Drosophila melanogaster*, which we reduced to 10,018 orthologous groups; proteins with significant similarity were considered as singletons in our processing, since paralogs may have arisen after speciation.

To determine the proteins in the *Drosophila *orthologous groups common to all insects studied here, called the *insect core set*, we used predicted proteins from insect genome sequences and EST sequences. We obtained 1346 orthologous groups from the intersection of the whole genomes of five *holometabolous *insects (see Methods). One aspect of our approximation is to use homologs to *Drosophila *proteins to characterize proteomes, implicitly assuming that function follows structure. This could contribute to differences in our characterization from the actual proteome, but it does not significantly detract from our use of the characterizations. We discuss further implications of our approximation in more detail below.

**Figure 1:**
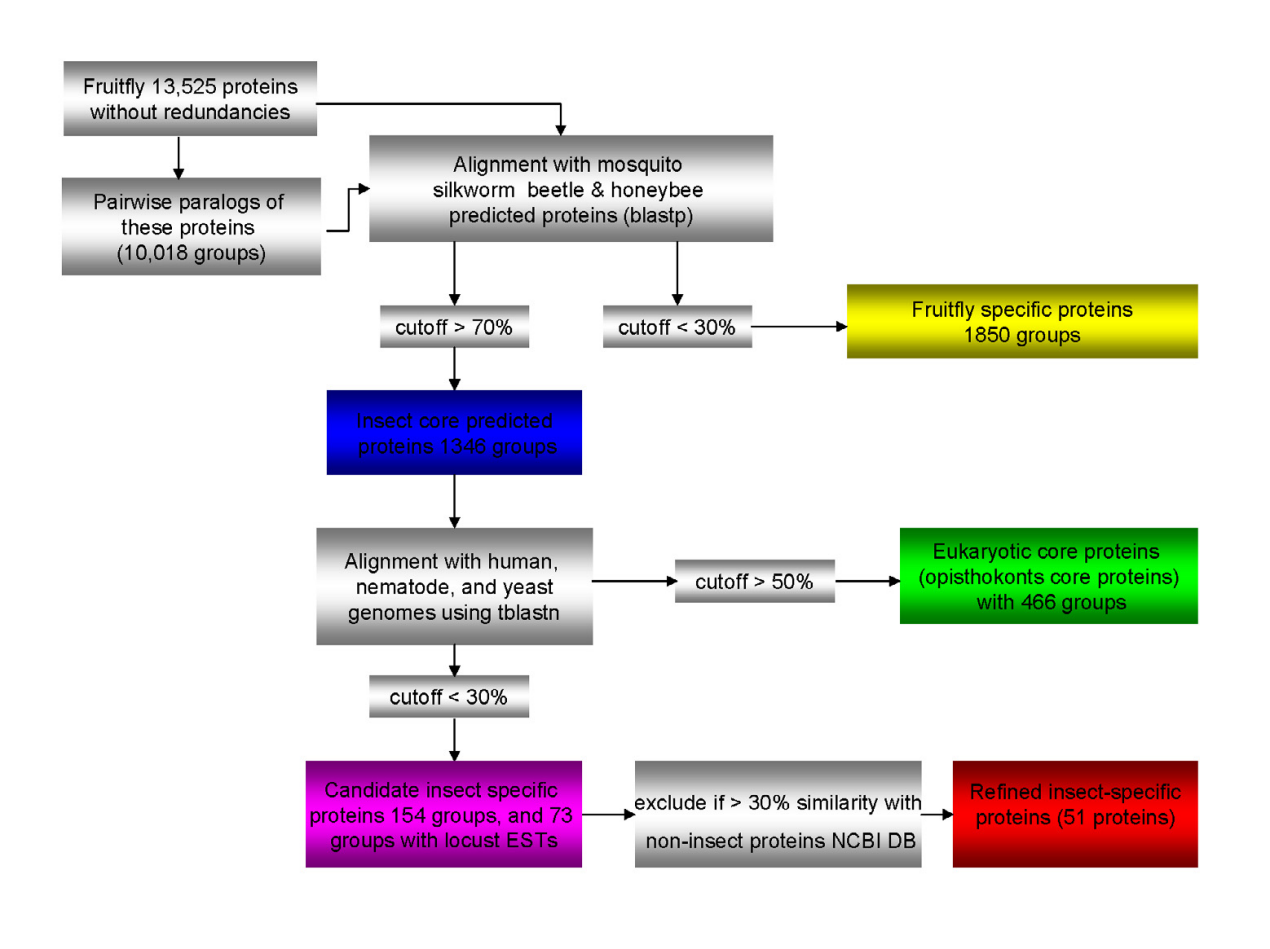
**Flowchart of computational analysis**. The pipeline was based primarily on genome comparisons; insect core proteins were distilled from four insects putative protein sets, and were searched against non-insect genomes to arrive at the insect-specific proteins and eukaryote/opisthokont core proteins. Also see Figure 2.

Using the insect-core protein set, we removed proteins with significant similarity to any genome sequence in yeast, human, and nematode (see Methods). The remaining 154 orthologous groups (with 360 proteins) form the *insect-specific set*, and 73 of these groups are represented in the *hemimetabolous *insect locust ESTs [see Additional file 1]. The insect-specific set contains proteins with homology evidence to all insects studied here; in addition, these sequences are without significant similarity to the non-insect species. Since we are interested in genes and proteins in insects which developed in insects after their divergence from other eukaryotes, we searched entire non-insect eukaryotic genomes in alignments with the insect-core proteins in order to exclude remnants of common ancestral genes. To refine the insect-specific proteins, we removed proteins with similarity to non-insect proteins in the NCBI protein database as described in Methods (Figure 1). This reduced the 360 candidate insect-specific proteins to the final insect-specific set consisting of 51 proteins [see Additional file 2].

We found 466 proteins with homology to all eukaryotes considered in this study using methods similar to those above [see Additional file 3].

**Figure 2:**
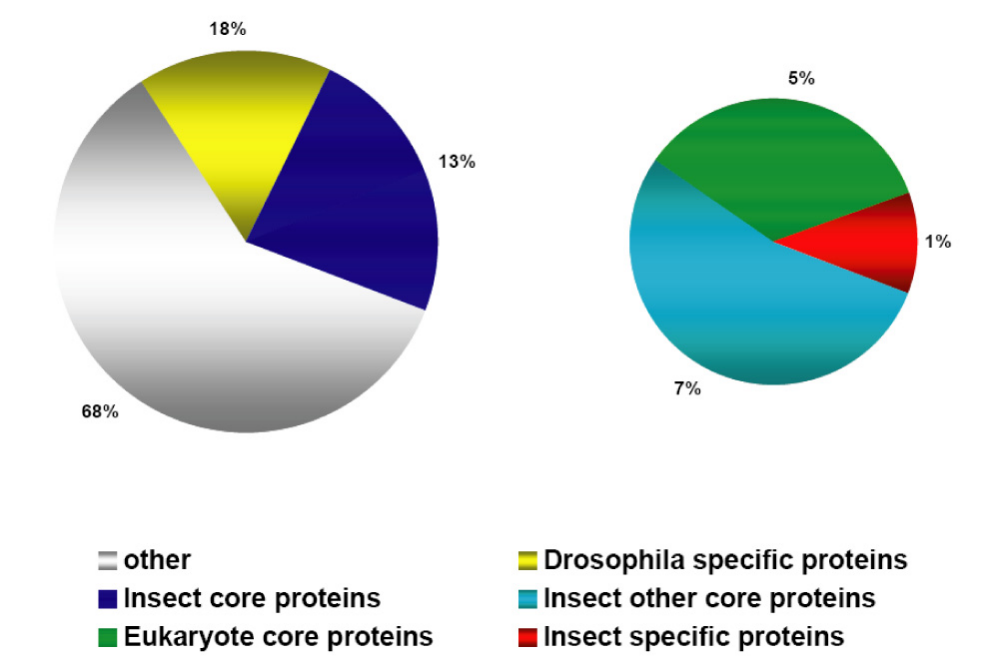
**Clustering *Drosophila *proteins**. *Drosophila *proteins were clustered into paralogous groups based on their sequence similarity. Using methods described in the text, 1850 groups of *Drosophila *specific proteins make up 18% of fruitfly paralogous groups, and 1346 (13%) insect core proteins were identified. In the insect core set, 466 groups (5%) can be found in other eukaryotes, and 154 groups (1%) are insect specific.

As the eukaryotes used in this study are all opisthokont, this set of proteins should be properly considered opisthokont core proteins. Many of these eukaryotic core proteins – the opisthokont core proteins – are involved in housekeeping or general metabolic processes. We also defined 1850 proteins as *Drosophila *specific by eliminating proteins homologous to other insect proteins as discussed in Methods (Figure 2).

### GO annotations and functional categories

We categorized proteins in the eukaryote (466 groups in opisthokont) and insect-specific sets (154 groups) using high-level gene ontology categories with results shown in Figure 2. In both the eukaryote and insect-specific sets, metabolic proteins constituted the highest fraction, 25% and 20%, respectively. Disproportionately represented categories are interesting to consider for candidate proteins that confer distinguishing characteristics. In the eukaryote/opisthokont set, genes responsible for processes such as cell division, cell motility, cell cycle, reproduction and cellular process are more highly represented by factors from about two to twenty. These proteins and their respective functional categories may distinguish insects less from eukaryotes/opisthokont than those proteins in categories that have a significant representation in the insect-specific set and are underrepresented in the eukaryotic/opisthokont set. These more highly represented categories in the insect-specific set are: larval development (2% in opisthokont, 4% in insect); defense response (0 in opisthokont, 6% in insect); and stress respone (0.2% in opisthokont, 6% in insect). What's more, a significant number of the insect-specific proteins were found to be related to pheromone/odorant binding proteins (OBP), insect cuticle proteins, and proline-rich proteins [see Aditional file 2].

## Discussion

### Biological process categories

**Figure 3:**
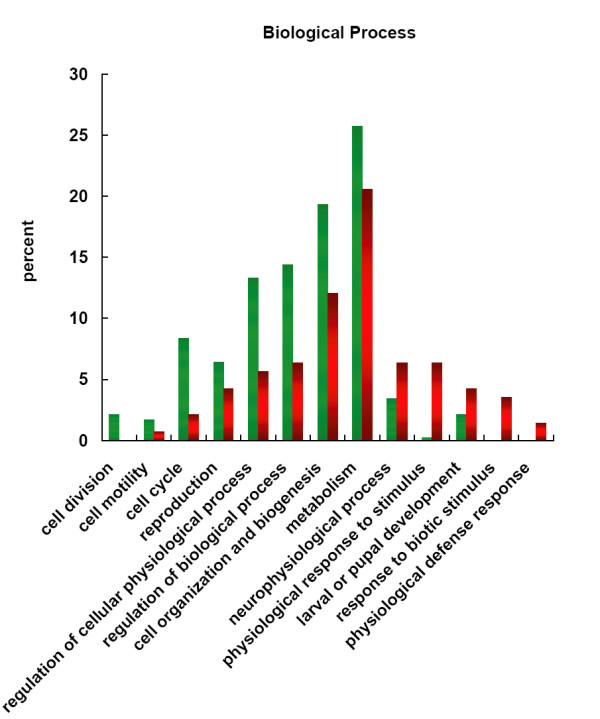
**Gene Ontology classifications**. Classification of insect specific proteins and eukaryote/opisthokont core proteins according to the *biological process *characterizations of the Gene Ontology System. Eukaryote/opisthokont core proteins are graphed with green bars and insect-specific proteins are shown with red bars. Plots show percentage differences for each category.

Our analysis of the *eukaryote/opisthokont core *and *insect-specific *protein sets was based on functional categories representative of high-level GO designations. Metabolism is the largest category of our eukaryotic/opisthokont core and of the insect-specific proteins. Significantly larger categories for the insect-specific proteins relative to the eukaryote core are stimulus and defense response (Figure 3.). A representative insect-specific gene in the stimulus response category is PedIII/CG11390 which has been reported to function in sensory perception [9]. In the eukaryote/opisthokont core proteins, the more highly represented insect-specific categories are not pronounced fractions thereby highlighting the insect-specific proteins as candidates for specialized roles. In the eukaryote/opisthokont core, other housekeeping processes such as cellular division, cell cycle and cellular organization processes constitute a larger fraction of the total protein set. The disproportionate distribution of the eukaryote/opisthokont core and insect-specific sets may be at the very foundation of insect evolution. It is important to note that the disproportionate distributions of functional types of proteins between insects and eukaryotes/opisthokont may be caused to some degree by the methodology; the small number of proteins in the insect-specific core may be caused by the limited number of insect genomes used, artificially underrepresenting the insect proteome. However, assuming an approximately representative distribution of unrepresented proteins makes it unlikely that the overrepresented categories are invalid.

The five insects with whole genomes are all holometabolous and might not be representative of all insects. At present, a complete genome sequence for hemimetabolous has not been sequenced, most likely because hemimetabolous insects often have large genomes (more than 2 gigabases) [10]. Fortunately, 45,474 high quality EST sequences from the hemimetabolous insect *migratory locust *permit us to perform analysis with all insects [11]. We determined the insect-specific orthologs in the locust ESTs to arrive at a collection of six sets of insect-spectific proteins. Our analysis found the functional distribution of the orthologous proteins in of the six insects to be similar with the functional distribution of the largest set from the five holometabolous insects [see Additional file 2].

We have noted above, the computed insect-specific protein dataset is an approximatation dependent on available genome sequence. Inclusion of additional genomic data could alter the protein set. The lack of many representative outgroups might causes false positives, i.e. some proteins might be inaccurately included in our list. For example, the gene CG6895 related to immune function is identified as an insect-specific gene in this study, but its homolog was recently reported in the sea urchin [12]. Improved quality of genome sequences and gene annotations for the insects used in this study will improve the accuracy of our computed proteins sets [13,14].

### Molecular function categories

A considerable number of the 51 insect-specific proteins were found to be related to insect cuticle proteins and pheromone/odorant binding proteins (OBP) [see Additional file 2]. Molting and metamorphosis are crucial processes in the developmental history of the insects involving cuticular proteins. Cuticular proteins are involved in important composite structural materials for insect cuticles, which provide protection, support, and locomotion; these prevent water loss via a wax layer, provide sites for waste product deposition, and protect from ultraviolet radiation [15]. Olfaction is essential to insect survival and reproduction, such as in location of food sources and mate selection. These olfactory driven behaviors contribute significantly to the ability of insects to adapt to the environment. The odorant-binding proteins, which compose the insect olfactory system, are involved in the recognition of odorants of plants by insects [16,17]. The pheromone binding proteins (PBP), abundantly present in the sensillum lymph of pheromone-responsive antennal hairs, are thought to be important in the recognition and discrimination of species-specific pheromones [18,19]. The olfactory system in insects evolved as a remarkably selective and sensitive system, approaching the theoretical limit for a detector. Even a single pheromone molecule is enough to elicit impulses at the olfactory neuron [20,21]. The large number of odorant and olfactory proteins in the insect-specific set suggests that in the evolution and diversification of insects, communication and adaptation with the environment played key roles in shaping their morphological and physiological characteristics.

Other insect-specific proteins in our insect-specific set have been found essential to development through experimental procedures [22-25], supporting our insect-specific proteome characterization. Moreover, these have been found to be active in insects and are of interest for evolutionary reasons including their suspected roles in diversification. For example, the gene *sinuous *(CG10624), which is active in tracheal system development, can partially rescue the tracheal defects of sinuous mutants [22]. The *Exuperantia (Exu) *protein in our insect-specific set is the earliest factor known to be required for the localization of *bicoid *mRNA to the anterior pole of the *Drosophila *oocyte. *Exu *is highly enriched in the sponge bodies; mutation of *exu *in *Drosophila *may result in defection of embryonic development [23]. *Larval serum proteins *(*Lsp*), another type of protein in the insect-specific set, belonging to the hemocyanin superfamily. This family is thought to function as storage proteins that provide amino acids and energy during non-feeding periods of immature and adult development [24,25].

### Low mutation rate of insect-specific proteins

**Figure 4:**
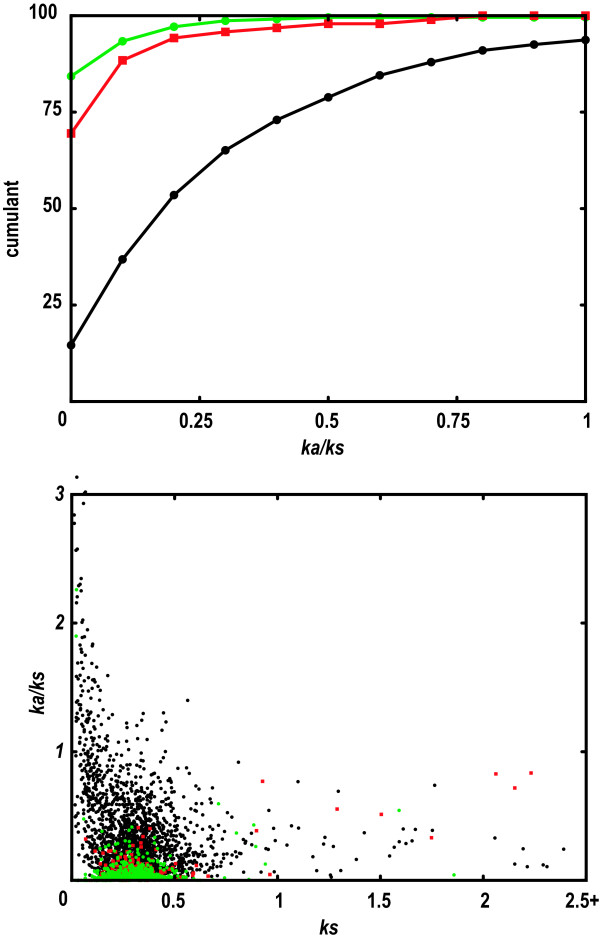
***Ka/Ks *distribution**. Nonsynonymous and synonymous substitution rates (Ka and Ks) were estimated for *Drosophila *specific, insect-specific, and eukaryote/opisthokont core proteins. *Drosophila *specific proteins are shown in black, insect-specific proteins in red and eukaryote/opisthokont core proteins in green. (a) Cumulative percentage of *Ka/Ks *ratios; (b) *Ka/Ks *versus *Ks *ratios.

It is widely accepted that all insects have arisen from a common ancestor that diverged from an aquatic arthropod more than 390 million years ago, and that they coevolved with a specific plant group [26]. Homologs to the insect-specific proteins should be present in the ancestor and be conserved by natural selection. To test this, we analyzed the ratio of the number of nonsynonymous substitutions per nonsynonymous site (*Ka*) to the number of synonymous substitutions per synonymous site (*Ks*) for the insect-specific proteins in *Drosophila*; in this analysis eukaryote/opisthokont core proteins and *Drosophila *specific proteins were used as controls. The high percentage of insect-specific proteins have a *Ka/Ks *ratio lower than 0.5 (Figure 4) suggesting negative selection in these proteins [27]. As non-synonymous changes are more likely to be deleterious, under negative or purifying selection pressure, these substitutions were eliminated in functionally active proteins, which may have provided a steady protein complement for insects [28]. Furthermore, the higher *Ka/Ks *ratio of insect-specific proteins is on average greater than that of the eukaryote/opisthokont core proteins. This may reflect the later appearance of insect-specific set,relative to proteins in the common eukaryote ancestor.

**Figure 5:**
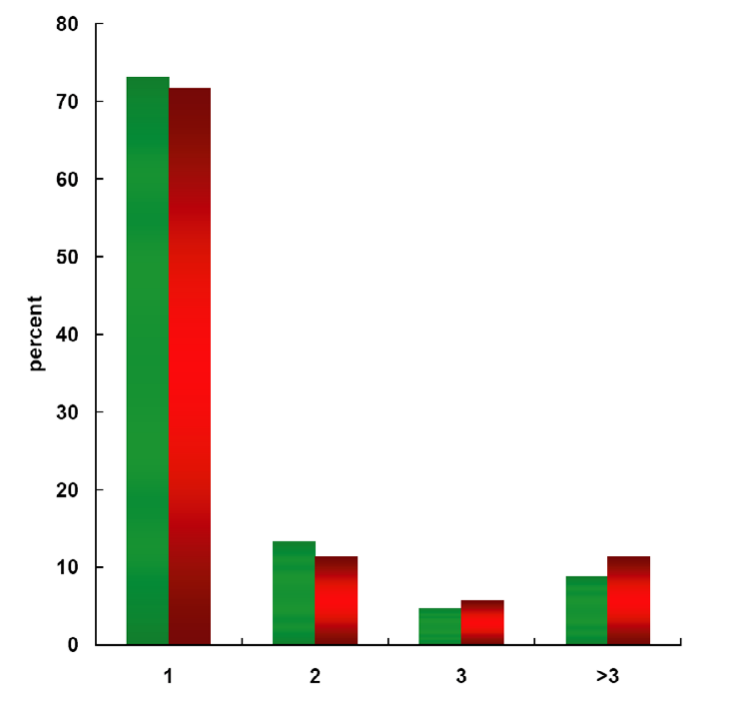
**Copy numbers of insect-specific proteins and eukaryote/opisthokont core proteins**. This plot shows the distribution of proteins by copy numbers of insect-specific proteins and eukaryote/opisthokont core proteins, insect-specific proteins in red and eukaryote/opisthokont core proteins in green.

To determine whether these conserved genes appeared with low redundancy, we ascertained the number of paralogs in the insect-specific genes with the number of paralogs in the eukaryote/opisthokont core genes. Gene duplication is considered one of the principal mechanisms in generating new genes and redundant sequences of genes with the same function [28]. Duplicated sequences of established genes often degrade to pseudogenes because purifying selection preserves essential coding sequence, while non-essential duplicates may lose function through random mutations favorable to natural selection. The relationship between duplicates and their functional ancestor is not fully understood. Some authors suggest that the stronger selective constraints on housekeeping genes relative to tissue specific genes is not due to their lower genetic redundancy [29]. However, our results agree with the observation of constrained duplication since most of the eukaryote/opisthokont core and insect-specific proteins are without paralogs (Figure 5). This suggests that genes with established function may tend to avoid duplication, thereby tolerating fewer genetic perturbations. However, the insect-specific proteins are inclined to arise from genes producing a greater number of paralogs, which is in contrast to proteins in the eukaryote/opisthokont core. This may confer insect adaptation to changes in the environment. For example, CG16799 and CG6421 have been found to function in defense response; both arise from paralogous groups in *Drosophila *with ten and four members, respectively.

Our analysis suggests that our working set of insect-specific proteins had been shaped by strong natural selection, with environment as one of the selective influences.

## Conclusion

An analysis of the genetic basis of evolution and development in insects was performed by characterizing the *eukaryote/opisthokont core *and *insect-specific *proteomes through genome analysis. Studies of the conservation and divergence between different organisms can provide clues to the molecular basis of species diversity and adaptation. The characterization of proteomes based on genome sequences provides a rapid method to approximate and update putative proteomes as genome sequences become available. Using this approach, we isolated fifty insect-specific proteins, many supported by experimental studies.

Proteins related to stress and immune responses constitute a significantly larger fraction of the proteins in our characterization of the insect-specific proteome, in contrast to our characterization of the eukaryote/opisthokont core proteome. The large component of olfaction and cuticle development proteins specific to the insect suggests the significance of communication and adaptation to the environment in insect evolution. Purifying selections in the evolution of insects were indicated in the analysis of nonsynonymous-to-synonymous substitution ratios, with a larger fraction of multi-paralog proteins possibly providing insects with an adaptive advantage over other eukaryotes. Due to the nature of our computatational method, our insect-specific proteins can increase or decrease with the inclusion of additional genome data from insects and non-insect species.

## Methods

### Sequence data

The protein sets in this work were founded on 18,282 protein sequences of *Drosophila melanogaster *[30] obtained from Ensembl [31]. Genes were predicted in genome sequences for *Anopheles gambiae *(mosquito) [32] and *Bombyx mori *(silkworm) [33,34]. Proteins of *Tribolium castaneum *and *Apis mellifera *[35] were obtained from HGSC[36]. Homologs to the insect protein sequences were isolated in annotated genomes of human [37], yeast [38] and nematode [39]. We obtained the *Anopheles gambiae *(mosquito) genome annotated with 16112 proteins (anopheles-21.2b) from Ensembl. The annotated human genome sequence draft (hg17) was obtained from UCSC [40], the worm genome (celegans-21.116a) from Ensembl, and the yeast genome from Saccharomyces Genome Database SGD [41]. Proteins where obtained for *D. yakuba *from FlyBase for use in *Ka/Ks *analysis. The locust (*Locusta migratoria*) UniGene collection with 12,161 ESTs and cDNA sequences was obtained from LocustDB [11,42].

### Sequence analysis

Sequence alignment was performed with BLAST [43] using the BLOSUM62 scoring matrix and default parameters. Gene prediction was performed using the gene-finder algorithm *BGF *used in BGI GeneFinder [44] based on *GenScan *[45] and *FgeneSH *[46].

### Paralog definitions

We grouped homologous protein sequences into *paralogous groups*. Protein sequences were considered paralogous if their alignment had an E-value less than or equal to 1e-5 and the alignment covered 70% or more of one of the aligned proteins. We represented paralogous groups by the longest member in the group, with the size of the group determined by the number of unique sequences in it.

### Proteome characterizations using genomic based pipeline

We defined protein sets based on *Drosophila *proteins in our processing pipeline to characterize proteomes. Similarity with genome sequences, predicted proteins, and ESTs was used to cull sets determined in the processing pipeline as described below. Thus, it is important to note that the various protein sets we computationally arrive at characterize insect and eukaryote proteomes through homology.

The insect core set was arrived at by selecting proteins in the *Drosophila *protein data set with similarity to mosquito and silkworm protein sequences predicted by genome analysis, and with similarity to the locust EST sequence data. Protein sequences for predicted genes in silkworm and mosquito were aligned against fruit fly using blastp [43] and considered homologous with an E-value cutoff of 1e-5 or less; in addition, we required that the length of the aligned sequences be within 70% of each other (Figure 5).

The insect-specific protein set was derived from the insect core set, where proteins *without *significant alignment to the genome sequences of human, nematode, or yeast were included (E-values of 1e-5 or less). In addition, sequences in the insect core set were retained for the insect-specific set if any alignment covered less than 30% of the insect protein sequence. The insect-specific proteins were further assessed against the NCBI protein database, retaining sequences without significant similarity and less than 30% alignment coverage with all non-insect proteins (Figure 5).

Proteins in the insect core set with an E-value cutoff of 1e-5 or less in alignments with each of the non-insect eukaryotes, and involving 50% or more of the insect protein in the alignments, were included in the eukaryote core protein set.

### Interpro annotation of insect proteins

Functional annotations for proteins in each of the working insect proteomes were determined using the annotation tool *Interproscan *[47] and Gene Ontology nomenclature [48]. GO terms were downloaded from Gene Ontology Consortium.

### *Ka/Ks *ratio calculation

We selected the most similar orthologs to *Drosophila melanogaster *in the *Drosophila yakuba *proteome, YN00 [49], to calculate *Ka/Ks *ratios.

## Authors' contributions

GJZ carried out the sequence alignment, analysis of the data, drafting and revision of the manuscript. HSW participated in the study concept and drafted the manuscript. SJJ participated in the acquisition of data and sequence alignment and drafted the manuscript. WXL and ZHK participated in sequence alignment. GKSW, JW and LK conceived of the study and participated in its design. WW, TC and LK critically revised the manuscript and assessed results. All authors read and approved the final manuscript.

## Supplementary Material

Additional file 1 **Insect-specific proteins from five whole genomes of insects**. Proteins homologs in the five whole insect genomes were listed in this table. Click here for file

Additional file 2 **Refined insect-specific proteins**. The refined 51 insect-specific proteins are listed in the table with *Ka/Ks*, Interpro annotations, GO terms, mutant phenotypes, and homologs with other insects. GO terms were downloaded from the Gene Ontology Consortium. Mutant phenotypes were downloaded from FlyBase. Proteins with significant mutant phenotypes are highlighted in red. Click here for file

Additional file 3 **Eukaryote/opisthokont core proteins**. A list of 466 eukaryote/opisthokont core proteins with horologes in five insects was presented. Click here for file
